# Intrafollicular and Systemic Dopamine, Noradrenaline and Adrenaline Concentrations in Cycling Mares

**DOI:** 10.3390/ani10101896

**Published:** 2020-10-16

**Authors:** Katiuska Satué, Esterina Fazio, Maria Dolores Rubio, Cristina Cravana, Pietro Medica

**Affiliations:** 1 Department of Animal Medicine and Surgery, Faculty of Veterinary Medicine, CEU-Cardenal Herrera University, Tirant lo Blanc, 7, Alfara del Patriarca, 46115 Valencia, Spain; 2 Department of Veterinary Sciences, Veterinary Physiology Unit, Messina University, Via Palatucci, 98168 Messina, Italy; fazio@unime.it (E.F.); ccravana@unime.it (C.C.); pmedica@unime.it (P.M.); 3 Department of Cellular Biology, Physiology and Immunology, Faculty of Veterinary, University of Córdoba, Campus of Rabanales, 14071 Córdoba, Spain; ba1rulum@uco.es

**Keywords:** adrenaline, cycling mare, dopamine, follicular fluid, noradrenaline

## Abstract

**Simple Summary:**

This study provides new evidence on the physiological changes of catecholamines in follicular fluid during the follicular growth in the mare. Both dopamine and epinephrine increase in the follicular fluid with the advance of follicular development, although norepinephrine decreases. These changes could be related to the existence of systemic, autocrine and/or paracrine mechanisms of synthesis, metabolism and interconversion of catecholamines for the regulation of follicular growth and development.

**Abstract:**

In some species, catecholamines in follicular fluid (FF) are related to local physiological events responsible for the regulation of ovarian functions and oocyte maturation. The aim of the present study was to determine and compare intrafollicular and systemic concentrations of dopamine (DA), noradrenaline (NA) and adrenaline (AD) in cycling mares. Sixty ovaries were collected during breeding season from 30 mares raised for slaughterhouse meat production, with clinically normal reproductive tracts, were evaluated. Blood samples were collected prior to slaughter. Follicles were classified into three categories in relation to size: small (20–30 mm; *n* = 20), medium (≥31–40 mm; *n* = 20) and large (≥41 mm; *n* = 20). Follicular fluid (FF) samples were extracted from each follicle. Intrafollicular DA, NA and AD concentrations were significantly higher than the systemic concentrations (*p* < 0.05). Intrafollicular DA concentrations were higher in medium than small and large follicles (*p* < 0.05). Intrafollicular NA concentrations were higher in small than medium and large follicles (*p* < 0.05). Intrafollicular AD concentrations were higher in large than small and medium follicles (*p* < 0.05). Follicle diameter was significantly and negatively correlated with NA and AD (*p* < 0.05). A significant correlation of the same hormone concentration in FF and in systemic fluid was observed (*p* < 0.05). In summary, the FF can serve as an intraovarian catecholamine-storing compartment, with the ability to release neurotransmitters in a regulated way. These results provide novel insights into the neuronal nature of the follicle, suggesting the involvement of catecholamines in normal ovarian functions in mares.

## 1. Introduction

In mares, the involvement of dopamine (DA), noradrenaline (NA) and adrenaline (AD) on reproductive physiology is documented, since the catecholamines regulate gonadotropin-releasing hormone (GnRH), follicle-stimulating hormone (FSH), luteinizing hormone (LH) and prolactin (PRL) secretion [1,2,3]. Furthermore, the administration of DA antagonists stimulates the follicular recrudescence in anestrus mares [4,5,6,7], although they are not always capable of maintaining a sustained increase of PRL [5,8]. This fact suggests that a local ovarian mechanism, involving PRL or DA, affects the seasonal follicular growth. High concentrations of DA were found in the antral fluid of preovulatory follicles in human [9,10,11,12,13,14,15] and rat [16].

In pig’s follicular fluid (FF), NA concentrations increase significantly during the follicular phase, with physiological implications in preovulatory events, as during luteinization [17]. Fernández-Pardal et al. [18] documented that small follicles contain greater concentrations of NA than medium follicles. However, significantly greater concentrations of both AD and NA were found in the FF of large preovulatory follicles compared to medium-size follicles. In addition, Kozłowska et al. [19] showed that in the porcine ovaries’ cystic fluid NA was lower as compared to medium follicles of the control group. The contents of DA and AD in the cystic fluid were below the threshold of detection. After dexamethasone (DXM) injections, the concentration of NA in the FF from small follicles was greater than in the control group. The concentrations of DA in FF from small follicles was similar in the control and DXM groups. Moreover, the content of AD in the FF from small follicles in both groups, as well as the concentrations of DA and A in the FF from medium follicles of the control group, were below the threshold of detection. In humans, NA concentrations in FF were substantially higher than those in plasma samples, and a positive correlation between FF and plasma concentrations was found [13].

Although DA was detected in the FF of mare follicles of all sizes, NA has not been identified in medium or large follicles [20]. It was presumed, although not verified, that cortical ovarian samples contained primordial and/or primary follicles. The results indicated that both dopaminergic receptor type 2 (DA D2r) and follicle-stimulating hormone receptor (FSHr) mRNAs exist in similar quantities in the cortex of mare’s ovaries during winter anestrus and summer cyclicity. This suggests that DA does not influence follicular growth through FSHr production. However, the amount of DA reaching the cortical DA D2r receptors could be rapidly modulated through dopaminergic innervation of these areas.

From the clinical point of view, the study of FF has an immense value for better understanding the regulatory mechanisms of fertility in female reproduction. Considering that FF is a compartment with the ability to store and release catecholamines and that it could serve as a reserve to maintain a higher availability of these molecules, the hypothesis of this study was to verify the existence of catecholamines in FF for their possible contribution by systemic sources in mares. On this basis, the objective of the present study was to determine DA, NA and AD concentrations in FF and blood samples, by taking into account the correlations between molecules and the possible contribution of systemic catecholamine concentrations to FF contents.

## 2. Materials and Methods

### 2.1. Animals

All procedures involved in this study were approached by the CEU-Cardenal Herrera University Committee of Ethics in Animal Research and were carried out by considering the RD 37/2014 that regulates the protection of animals at the time of slaughter and the EU directive 2010/63/EU.

The study was conducted in the northern hemisphere during months of the breeding season (April and May 2018). During this period, the ambient temperature ranged from 27–31 °C, with a relative humidity of 40–60%. A total of 30 clinically healthy mares (local autochthonous mares for meat production, which mainly include Draft, Hispano–Breton and related crosses) aged 6.6 ± 1.3 years were studied. According to system described by Henneke et al. [21], the animals had a body condition score (BCS) from 7 to 8 out of 9, presenting a mean weight of 533 ± 7.3 kg. All animals were subjected to the same management and feeding conditions, represented by orchard grass–alfalfa mixed hay and had free access to mineral salt and fresh water in a sheltered area. The slaughter was localized in Valencia (Spain), with geographic coordinates of latitude: 39° 31′ 0.01″ N and longitude: 0° 25′ 0.01″ E. The official veterinarians for each stockyard and slaughterhouse accepted responsible participation in the study, and only mares with a reproductive history of normality in their estrus cycles were included in the study. The veterinary examination of the animals prior to slaughter consisted of careful review of official documentation, which included livestock of origin, sanitary registration number, suitable health status, deworming and vaccination plan, and clinical and reproductive history of the animals, along with clinically normal reproductive tracts after slaughter. The inclusion criteria for the animals were (1) absence of reproductive diseases in the clinical examination; (2) absence of inflammatory processes or infections that had required treatment or hospitalization during the month prior to the onset of the study; (3) to be vaccinated and dewormed correctly and (4) to be younger that 15-year-old, have no conformation defects that affect the perineum and vulva; (5) normal involution of the uterus in previous births, and lack of previous history of reproductive diseases that affect fertility.

### 2.2. Collection of Blood and Ovaries

From each animal blood samples (20 mL) were collected from the jugular vein 1 h before slaughtering (gunshot), while mares were in lairage, a procedure that took just a few seconds for each horse. Blood samples were collected using evacuated tubes (Venoject, Terumo^®^; Belgium) and were transferred into a polypropylene tube containing EDTA (1 mg/mL of blood). Samples were centrifuged at 1200× *g* for 10 min, and plasma was collected and stored at 4 °C in portable coolers for later transport to the laboratory. After the slaughter, only the ovaries of the normal reproductive tracts were collected. The period between the slaughter and collection of ovaries in no case exceeded 2 h, as proposed by Hinrichs et al. [22]. All ovaries were placed in containers with 0.9% physiologic saline plus penicillin (100 IU/mL) and streptomycin (50 mg/mL) and were transported to the laboratory in individually labeled plastic bags in thermal containers (at 25 °C) [23].

### 2.3. Collection of Follicular Fluid

Ovaries were washed three times with sterile saline and the follicles were direct measured with a digital Vernier caliper and categorized according to diameter as small (20–30 mm; *n* = 20), medium (≥31–40 mm; *n* = 20) or large (≥41 mm; *n* = 20). FF was aspirated using a different sterile syringe and 22 G needle for each follicle. Following collection, the FF samples were centrifuged for 10 min at 1200× *g* to eliminate the cumulus oocyte complexes. Only the supernatant (pure FF) was removed and stored in 0.5 mL aliquots at −20 °C until subsequent analysis.

### 2.4. Catecholamine Assay

The systemic and intrafollicular DA (pg/mL), NA (ng/mL) and AD (ng/mL) concentrations were determined by competition EIA-Technical 3-CAt EIA (Demeditec Diagnostics GmbH, Germany) specifically validated for the equine species [24]. The percentages of recovery for DA, NA and AD in plasma were 90.0%, 97% and 92%, respectively, and in FF were 93.0%, 96% and 95%, respectively. The detection limits for DA, NA and AD concentrations were 5 ng/mL, 50 ng/mL and 10 ng/mL, respectively. Serial dilutions up to 1:64 of pooled plasma and FF samples showed ranges of 14.0–917 ng/mL, 1.3–81.4 ng/mL, and 4.9–339 ng/mL for DA, NA, and AD, respectively. The intra-analysis CVs for DA varied between 9.5% and 15.8%, for NA between 9.8% and 16.1%, and for AD between 6.9% and 15%. The inter-analysis CVs for DA ranged between 15.9% and 18.2%, for NA between 8.5% and 15%, and for AD between 13.2% and 15.4%.

### 2.5. Statistical Analyses

Descriptive statistics mean ± standard deviation (SD) for DA, NA and AD concentrations in FF of small, medium and large follicles and in blood plasma were calculated. Normality was verified in all the data groups, using the Kolmogorov–Smirnov test. To determine the magnitude of variation in the concentrations of these constituents of FF and plasma in follicles of different diameters, data were subjected to one-way ANOVA analysis. Post-hoc comparisons were performed using Tukey’s test. The relationship between FF and systemic DA, NA and AD concentrations was examined by linear regression analysis, and the correlation was expressed by Pearson’s correlation coefficient. Differences were considered statistically significant when *p* < 0.05.

## 3. Results

In all the samples, intrafollicular concentrations of DA, NA and AD were significantly higher than the systemic ones (*p* < 0.05), and are presented as mean ± SD.

**Figure 1:**
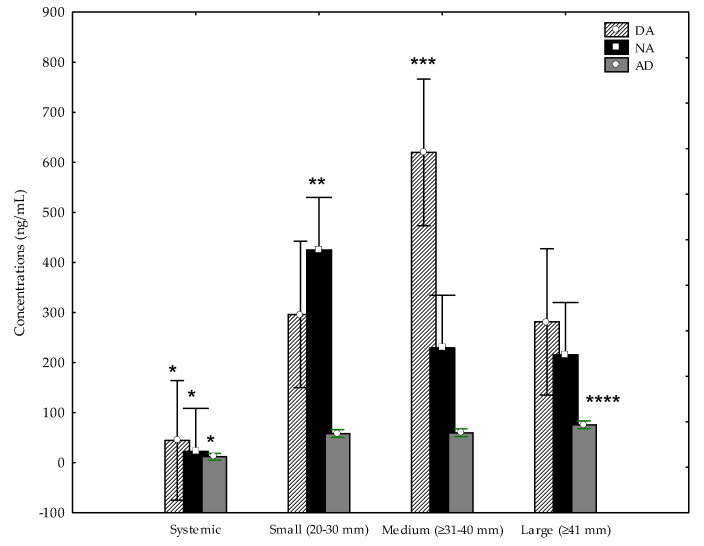
Concentrations of dopamine (DA), noradrenaline (NA) and adrenaline (AD) in systemic and follicular fluid (FF) of different follicles of mares (mean ± SD). Systemic fluid vs. small, medium and large follicles: * *p* < 0.05. Small vs. medium and large follicles: ** *p* < 0.05; medium vs. small and large: *** *p* < 0.05; large vs. small and medium **** *p* < 0.05.

Intrafollicular dopamine (DA) concentrations were higher in small (306.2 ± 113.8 ng/mL), medium (707.9 ± 360.6 ng/mL) and large (351.2 ± 132.5 ng/mL) follicles than systemic fluid (36.65 ± 6.45 ng/mL; *p* < 0.05). Medium follicles showed higher DA concentrations than small and large follicles (*p* < 0.05; Figure 1).

Intrafollicular noradrenaline (NA) concentrations were higher in small (472.2 ± 227.7 ng/mL), medium (244.7 ± 93.9 ng/mL) and large (210.1 ± 81.6 ng/mL) follicles than systemic fluid (17.12 ± 2.35 ng/mL; *p* < 0.05). Large follicles showed higher NA concentrations than small and medium follicles (*p* < 0.05; Figure 1).

Intrafollicular adrenaline (AD) concentrations were higher in small (48.9–83.2 ng/mL), medium (53.1–71.8 ng/mL) and large (47.7–93.3 ng/mL) follicles than systemic fluid (6.59–19.6 ng/mL; *p* < 0.05). Large follicles showed higher AD concentrations than small and medium follicles (*p* < 0.05; Figure 1).

Follicular diameter increased significantly in medium and large respect to small follicle sizes (*p* < 0.05) and was significantly and negatively correlated with NA and AD (*p* < 0.05).

A significant correlation of the same hormone concentration in FF and in systemic fluid was observed (*p* < 0.05; Table 1).

## 4. Discussion

### 4.1. Intrafollicular Catecholamine Concentrations in Mares/Humans/Other Species

The intrafollicular concentration of catecholamines in this study completely differed from those previously reported in the same species [20], despite the similarity in categorized follicle sizes (small: <25 mm; medium: 26–35 mm and large: >35 mm). Indeed, King et al. [20] observed no noradrenergic activity in the FF of medium and large follicles, showing an inverse relationship between follicular size and intrafollicular DA concentrations. Our results may indicate that DA and NA exercise a role in early follicular recruitment, but not in late antral follicular development; nevertheless, intrafollicular AD is present during the development of the dominant follicle until it reaches preovulatory size. Despite these differences, the dynamic shown by DA during the follicular development period was similar in both the studies. In fact, DA concentrations were significantly higher in large and medium than small follicles. The variability between these results may be due to the wide standard deviations, as well as the small number of follicles considered in these studies, manipulation of the samples, laboratory methods employed and differences in the metabolic activities within the follicles [25]. Indeed, diverse methods used to determination of catecholamines in FF as high performance liquid chromatography (HPLC) [13] or kits ELISA [14] different from the one used in this study could be related with the differences among results of diverse researchers.

Substantial intrafollicular catecholaminergic activity in mares suggests an extra- or intra-ovarian production or synthesis. In women, the FF of large follicles accumulates NA in concentrations higher than systemic ones and on similar form to mare, significant correlations between both fluids were obtained [25]. Although the mechanisms for accumulation of catecholamines in the FF remain unknown, in women undergoing treatment for in vitro fertilization, NA can be released from sympathetic fibers innervating the interiors of the follicles, interstitial gland and ovarian vasculature [25,26,27]. Contrary to the case in the mare, the NA in the FF in women [10,13,25] and cows [28] markedly increased in preovulatory follicles. These increases are produced in synergy with increased preovulatory release of NA from the nerve terminals in pigs [18] and humans [29] at the time of preovulatory gonadotropin surge. The synergistic action of catecholamines with gonadotrophins could favor the myocontractility necessary for expulsion of the oocyte at ovulation, as observed in cows [30] and women [29]. Although in the mare, the sources of catecholamines in the FF are unknown, experimental studies in women and in laboratory animals have shown different origins of these neurotransmitters. In women, the access of NA to the granulosa cells of larger follicles depends on diffusion through the basal lamina and granulosa cells layers during follicular growth. Once NA is taken up by granulosa cells, it is metabolized via mitochondrial monoamine oxidase A (MAO-A) within these cells. Furthermore, granulosa cells also take up and metabolize NA in humans [25] and rats [31].

### 4.2. Ovarian Synthesis, Uptake, or Interconversion Mechanisms of Catecholamines in Mares/Primates/Other Species According to Receptors’ Expression

Another point is that DA antagonist treatment of acyclic mares provides controversial results. Hence, some studies reported that treatment with sulpiride, domperidone or perphenazine stimulated ovarian recrudescence, advancing the first ovulation of the year in mares maintained outside [4,5,7]; other authors reported a stimulatory effect of sulpiride, but not of domperidone, in mares housed indoors only [32], or a stimulatory effect in photo-stimulated mares housed indoors [2,33]; finally, others authors have not observed an increase in ovulation [34]. Factors such as environment, photoperiod, temperature, or stress may influence the efficacy of these treatments [2,34,35]. The exact mechanism of action of catecholamines, DA in particular, on follicular dynamics is not known, but is believed to involve the regulation of prolactin as its pituitary production, which primarily is regulated through inhibition by the neurotransmitter DA. The DA antagonist increases the circulating prolactin and estradiol concentrations after the first week of treatment, indicating a follicular development as a result of inhibition of DA and increased stimulation of gonadotrophin receptors on the ovary [36]. However, the DA antagonists allow follicular development only in the presence of FSH secretion and the ability of the ovary to respond to them in the mare. Indeed, the mRNA expression for FSH receptors in the ovarian cortex was minimal during anestrus and increased approximately five-fold during the breeding season [37]. Moreover, the presence of catecholamines in the FF is intimately related to the expression of receptors in the ovarian follicle [25]. In mares, DA D2r receptors are highly expressed in the luteal tissue and ovarian cortex, but they are not highly expressed in granulosa and theca cells, while D1r receptors are only expressed in corpus luteum [20]. The low presence of DA receptors in granulosa cells makes the synthesis of this catecholamine decrease in the FF of larger-sized follicles. However, these findings do not support the idea that DA synthesis is exclusively mediated by the dopaminergic receptors, since in our study, both DA and NA considerably rise in the follicles of small and medium size, although they decrease later in the preovulatory follicles. The substantial variation observed in the mare could be related to the complex metabolism of catecholamines, since DA is the precursor of follicular NA and both can be metabolized. These findings suggest that the depletion of endogenous DA and NA in late follicular development stages could be a consequence of reduced synthesis and uptake, or of interconversion mechanisms of DA for the final synthesis of AD, with the aim of reaching the preovulatory stage.

Furthermore, NA targets β-adrenergic receptors of granulosa cells and elevates cAMP, allowing them to serve as catecholamine-storing cells within the ovarian follicle and ensuring that DA and/or NA are present in the granulosa cell compartment [31]. In addition, adrenergic nerves supply the theca and the evidence for DA secretion by nerve cells to the Graafian follicle has been shown in cows [28]. Furthermore, it has also been reported that DA-containing nerve terminals exist in the thecal cell layer and around the walls of mature follicles in guinea pigs, but not in the granulosa cell layer. However, granulosa cells also express transporters for NA and DA, favoring their intracellular storage [14,25,31,38].

Metanephrine, a metabolite of DA and NA in FF and granulosa cells, also provides clear evidence of the follicular metabolism of catecholamines [25]. In addition, a ligand (NA in FF) and its receptor (ADRB-2 in granulosa cells) co-exist in the large antral follicles of primates [39]. Likewise, NA can be synthesized by the neuron-like cells in the ovary and oocytes. Indeed, in non-human primates, oocytes, but no other ovarian cell types, express the enzyme DA hydroxylase (DBH), and under experimental conditions can take up its and convert it to NA [40]. Hence, a complex ovarian DA system, which includes receptor-mediated roles for DA and DA metabolism, may be assumed [14]. Since NA is the most abundant neurotransmitter released by the sympathetic nerves in most mammalian species [27], the granulosa cells take up and store it, releasing it upon depolarization. Although ovarian denervation inhibits the follicular growth, it reduces, but does not eliminate, NA in the gland [41], implying the existence of an additional source of catecholamine synthesis or participation of intraovarian cells in ovarian NA homeostasis.

### 4.3. Effects of Systemic and Follicular Fluid Catecholamines on the Reproductive Physiology in Mares

In summary, the presence of DA, NA and AD in the FF of the mare indicates the existence of interspecific differences regarding the content of catecholamines with respect to other species. The great amount of DA, NA and AD in FF could indicate the existence of systemic and autocrine and/or paracrine mechanisms related to catecholamine synthesis, metabolism and subsequent use, guaranteeing the successful growth and development of ovarian follicles. This physiological response could have pivotal applications in the reproductive clinic for the diagnosis of pathological processes that occur with infertility in the mare. However, future investigations would be required to clarify these complex mechanisms of interconversion and sources of catecholamines in the FF of mares.

## Figures and Tables

**Table 1:** Systemic and intrafollicular dopamine (DA), noradrenaline (NA) and adrenaline (AD) correlation coefficients in follicles of mares. Results are presented as mean ± SD; * *p* < 0.05.

Parameters	N (90)	r	Equation of Regression Line
DA (ng/mL)		0.64	Systemic/FF = 1.3557 + 0.99 *
NA (ng/mL)		0.67	Systemic/FF = 1.3410 + 0.00154 *
AD (ng/mL)		0.93	Systemic/FF = 0.90257 + 0.01575 *
